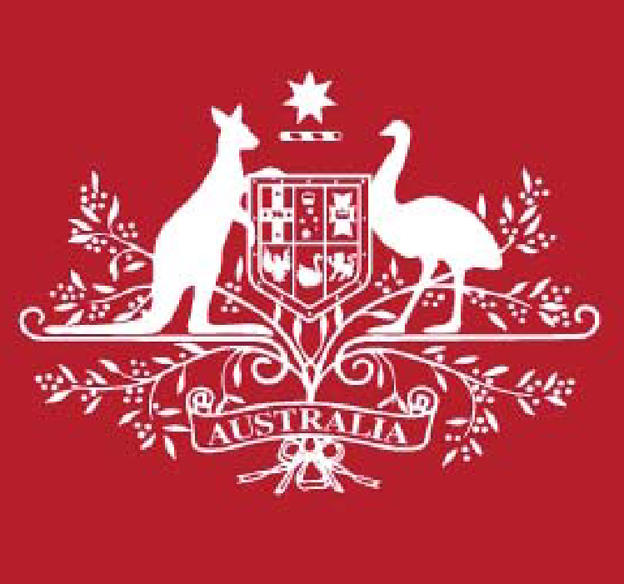# EHPnet: Australian Quarantine and Inspection Service

**Published:** 2007-07

**Authors:** Erin E. Dooley

As part of the Australian government’s Department of Agriculture, Fisheries, and Forestry, the Australian Quarantine and Inspection Service (AQIS) provides a host of essential services to help ensure that humans, animal and plant products, and even postal mail going to and from the country are healthy and do not pose a threat to Australia’s unique environment. Its almost 3,000 employees inspect and certify agricultural exports, as well as oversee quarantine controls at Australia’s borders. The AQIS website at **http://www.daff.gov.au/aqis/** provides an overview of these activities.

The Importing to Australia section of the site has subsections for the different types of items coming into the country, along with a subsection of general information. The Cargo Containers subsection addresses topics such as high-risk pests found in imported cargo. One survey found that 13% of the 14,500 import containers inspected between December 1997 and June 2000 contained contaminants of quarantine concern.

Quarantine measures help keep unwanted diseases and pests from entering Australia. One area of the country that is particularly vulnerable to these unwanted organisms is Northern Australia, which is separated from the islands of Southeast Asia by only very short distances. In 1989, the AQIS’s Northern Australia Quarantine Strategy (NAQS) was established to focus efforts on protecting this area. The NAQS employs scientific surveys and monitoring to provide early detection of incoming pests such as disease-carrying mosquitoes. The NAQS subsection of the AQIS site provides fact sheets and other publications as well as a list of exotic pests, weeds, and diseases targeted by the NAQS.

The AQIS uses X rays and “detector dogs” to screen around 180 million items of mail each year in its efforts to keep unwanted materials out of the country. About 80,000 high-risk items of mail are intercepted in these manners annually. A section of the AQIS site provides a rundown of what the screeners are looking for and informs people as to why certain items are targeted, even though they can be purchased in Australia. The categories of targeted products include dairy and egg products, seeds and nuts, plant material, plants and soil, and live animals and animal products.

The Quarantine section of the AQIS site also has a page on the detector dogs program. These beagles, employed on 75 teams, are trained to detect more than 30 different plant and animal items and work at international airports, mail centers, and private courier depots around the country.

Also in the Quarantine section is a subsection on pests and diseases. One portion of this section looks at four mosquito-borne diseases—dengue, malaria, West Nile fever, and yellow fever—while another focuses on four weed species. The four weeds pages describe the species and tell where each is currently established, how it spreads, and how its presence and spread could affect Australia. A directory of pest and disease fact sheets is also available.

## Figures and Tables

**Figure:**